# Half a Century of Wilson & Jungner: Reflections on the Governance of Population Screening

**DOI:** 10.12688/wellcomeopenres.16057.2

**Published:** 2020-08-17

**Authors:** Steve Sturdy, Fiona Miller, Stuart Hogarth, Natalie Armstrong, Pranesh Chakraborty, Celine Cressman, Mark Dobrow, Kathy Flitcroft, David Grossman, Russell Harris, Barbara Hoebee, Kelly Holloway, Linda Kinsinger, Marlene Krag, Olga Löblová, Ilana Löwy, Anne Mackie, John Marshall, Jane O'Hallahan, Linda Rabeneck, Angela Raffle, Lynette Reid, Graham Shortland, Robert Steele, Beth Tarini, Sian Taylor-Phillips, Bernie Towler, Nynke van der Veen, Marco Zappa

**Affiliations:** 1 Science, Technology and Innovation Studies, University of Edinburgh, Edinburgh, EH1 1LZ, UK; 2 Centre for Biomedicine, Self and Society, University of Edinburgh, Edinburgh, EH8 9LN, UK; 3 Institute of Health Policy, Management and Evaluation, University of Toronto, Toronto, Ontario, M5T 3M6, Canada; 4 Department of Sociology, University of Cambridge, Cambridge, CB2 1SB, UK; 5 University of Leicester, Leicester, UK; 6 Children's Hospital of Eastern Ontario, Ottawa, Canada; 7 Melanoma Institute Australia, Wollstonecraft, Australia; 8 Kaiser Permanente Washington Health Research Institute, Seattle, USA; 9 University of North Carolina, Chapel Hill, USA; 10 National Institute of Public Health and the Environment, Bilthoven, The Netherlands; 11 Independent Scholar, Durham, NC, USA; 12 Danish Health and Medicines Authority, Kobenhavn, Denmark; 13 INSERM, Paris, France; 14 Public Health England, London, UK; 15 UK National Screening Committee, London, UK; 16 Ministry of Health, Mount Victoria, New Zealand; 17 Cancer Care Ontario, Toronto, Canada; 18 Consultant in Public Health, Bristol, UK; 19 Dalhousie University, Halifax, Canada; 20 University Hospital of Wales, Cardiff, UK; 21 University of Dundee, Dundee, UK; 22 University of Iowa, Iowa City, USA; 23 University of Warwick, Warwick, UK; 24 Department of Health and Ageing, Canberra, Australia; 25 Instituto per lo Studio e la Prevenzione Oncologica, Firenze, Italy

**Keywords:** screening, governance

## Abstract

**Background:** In their landmark report on the “Principles and Practice of Screening for Disease” (1968), Wilson and Jungner noted that the practice of screening is just as important for securing beneficial outcomes and avoiding harms as the formulation of principles. Many jurisdictions have since established various kinds of “screening governance organizations” to provide oversight of screening practice. Yet to date there has been relatively little reflection on the nature and organization of screening governance itself, or on how different governance arrangements affect the way screening is implemented and perceived and the balance of benefits and harms it delivers.

**Methods:** An international expert policy workshop convened by Sturdy, Miller and Hogarth.

**Results:** While effective governance is essential to promote beneficial screening practices and avoid attendant harms, screening governance organizations face enduring challenges. These challenges are social and ethical as much as technical. Evidence-based adjudication of the benefits and harms of population screening must take account of factors that inform the production and interpretation of evidence, including the divergent professional, financial and personal commitments of stakeholders. Similarly, when planning and overseeing organized screening programs, screening governance organizations must persuade or compel multiple stakeholders to work together to a common end. Screening governance organizations in different jurisdictions vary widely in how they are constituted, how they relate to other interested organizations and actors, and what powers and authority they wield. Yet we know little about how these differences affect the way screening is implemented, and with what consequences.

**Conclusions:** Systematic research into how screening governance is organized in different jurisdictions would facilitate policy learning to address enduring challenges. Even without such research, informal exchange and sharing of experiences between screening governance organizations can deliver invaluable insights into the social as well as the technical aspects of governance.

## Disclaimer

The views expressed in this article are those of the author(s). Publication in Wellcome Open Research does not imply endorsement by Wellcome.

## Introduction

Just over 50 years ago, the World Health Organization (WHO) published a landmark report on the “Principles and Practice of Screening for Disease” by James Maxwell Glover Wilson and Gunnar Jungner. The report was prompted by the growing use of medical tests to identify potential disease in apparently healthy individuals, and by a dawning awareness that the adoption of such “deceptively easy” tests could cause harm without necessarily providing benefit to those tested ^1^. The report accordingly sought to clarify the circumstances under which screening could be considered appropriate as a means of secondary prevention. Over the ensuing half century, the practice of screening has continued to evolve, using a growing range of technologies to test for more and more conditions, from rare genetic disease screening in newborns to cancer screening in adults. Throughout that period, Wilson and Jungner’s report has remained a key point of reference for assessing the appropriateness of screening—in particular their ten “principles” which specified critical preconditions for pursuing screening ( Box 1).

**Box 1. Wilson and Jungner’s Principles of Screening ^1^**

1. The condition sought should be an important health problem.

2. There should be an accepted treatment for patients with recognized disease.

3. Facilities for diagnosis and treatment should be available.

4. There should be a recognizable latent or early symptomatic stage.

5. There should be a suitable test or examination.

6. The test should be acceptable to the population.

7. The natural history of the condition, including development from latent to declared disease, should be adequately understood.

8. There should be an agreed policy on whom to treat as patients.

9. The cost of case-finding (including diagnosis and treatment of patients diagnosed) should be economically balanced in relation to possible expenditure on medical care as a whole.

10. Case-finding should be a continuing process and not a ‘once and for all’ project.

As the title of their report makes clear, however, Wilson and Jungner appreciated that the *practice* of screening, including how screening is organized and how principles are enacted, is just as important for securing beneficial outcomes and avoiding harms as the formulation of the principles themselves. Accordingly, in the decades since their report was published, in a growing number of jurisdictions, a range of governmental and quasi-governmental organizations have been set up with the express aim of exercising oversight of screening practice, including reviewing screening interventions, issuing guidance, and in some cases establishing organized screening programs to oversee quality and coordinate access to screening-related services. Within their respective spheres of influence, these “screening governance organizations”, as we designate them, have worked hard to promote beneficial screening practices and avoid attendant harms.

In developing their procedures, screening governance organizations have given considerable thought to the role of clinical and epidemiological evidence in assessing the merits of specific screening interventions. Screening decisions do not depend on evidence alone, however. They are also shaped by the circumstances under which decision-making takes place, including how screening is organized, but also—and crucially—how governance itself is organized and practised. This is apparent in the diversity of governance organizations that exist in different jurisdictions, in their different powers and remits, and in the often markedly different screening regimes they oversee. Yet to date, there has been relatively little reflection on the nature and organization of screening governance itself, or on how different governance arrangements may affect how screening is implemented, how it is perceived, and the balance of benefits and harms it delivers.

This lack of reflection marks a significant gap in our understanding of screening and how it might best be implemented. Decisions about whether and how to screen may have political as well as practical repercussions. Not least, inter-jurisdictional variation in the availability and organization of screening can prompt difficult questions about the reasons for such variation, potentially undermining public trust in local and national arrangements and weakening the legitimacy of screening governance organizations. There is consequently a need for a closer look at screening governance, including how screening governance organizations differ in constitution and the powers they exercise, how those differences relate to variation in the scope and practice of screening, and how such organizations account for their decisions to governments, healthcare providers, clinicians and the public.

To address this need, in June 2018, an international group of screening experts from nine countries met in London for a two-day workshop to commemorate the 50 ^th^ anniversary of Wilson and Jungner’s report, to share and compare experiences of screening governance, and to ask: What have screening governance organizations achieved to date? What key governance challenges remain? And how might collective, cross-jurisdictional knowledge-sharing help to address those challenges?

This paper sets out some key issues that arose in the course of the workshop discussions. Rather than attempting to document all the data and perspectives presented at the workshop, it seeks instead to highlight key areas in which the participants agreed that governance challenges remained and where continuing reflection and development were needed. It considers achievements and ongoing challenges in three principal areas of screening governance: the assessment of the relative benefits and harms of screening; the organization of population screening programs; and the variations in governance arrangements arising in different contexts. It concludes, as did the workshop, that much could be gained through sustained engagement across jurisdictions and the creation of further opportunities for comparison, reflection and ultimately improvement of screening governance.

## An expert policy workshop

The workshop was planned and convened by Sturdy, Miller and Hogarth, prompted by conversations and questions that had arisen in the course of their respective researches into screening governance. The remaining workshop participants were recruited through personal contacts and snowballing recommendations. The aim was not to recruit a “representative” sample of international screening experts—if indeed such a thing were possible. Rather, we sought to create an opportunity to share, reflect on and learn from a diversity of experience regarding the achievements and challenges of population screening in different jurisdictions. To that end, we set out to bring together expert actors from a range of different national and regional screening governance organizations, plus a number of social scientists involved in researching screening policy and practice, while keeping numbers small enough to permit round-table discussion.

The workshop was organized into seven sessions, each devoted to a different aspect of screening governance in national and cross-national perspectives, and each including short reflective presentations by three or four participants followed by round-table discussion. A final round-table session considered the findings of the workshop and next steps, including the decision to write and publish the present paper.

Based on notes taken at the workshop, Sturdy, Miller and Hogarth prepared a first draft of the paper, which was then circulated to all participants with a request for corrections, clarification and suggestions for revision. After substantial redrafting, the paper was again circulated to all participants, leading to a third and penultimate draft. Following a further round of circulation and minor revisions, the final version was approved by all the listed authors. Dr Mary White, Chief of the Epidemiology and Applied Research Branch in the US CDC’s Division of Cancer Prevention and Control took part in the workshop, commented on drafts of this paper and agreed to be acknowledged as a named contributor but not as an author. Dr Alberto Gutierrez, a Partner with NDA Partners, who took part in the workshop but did not contribute to the preparation of this article, agreed to be named in that capacity.

The findings, conclusions, and views expressed in this paper are those of the authors individually and collectively. They do not necessarily represent the official positions of the organizations they work for. Nor do they claim to represent opinion among screening policymakers or practitioners more generally.

## Adjudicating the benefits and harms of population screening

Emerging at a time of burgeoning enthusiasm for non-communicable disease screening ^2^, Wilson and Jungner’s report anticipated benefits and highlighted potential harms. Assessing then-current efforts as “at a very early and comparatively primitive stage in the systematic detection and treatment of early disease” ^1^, the report made a compelling case for systematic, evidence-based evaluation of new screening interventions. That case has only been strengthened by 50 years of scrutiny, which has deepened understanding of the possible sources of harm (false positives, false negatives, over-diagnosis) and its many manifestations (physical, psychological, financial, social) ^3– 5^. As Angela Raffle, Anne Mackie and Muir Gray put it: “All screening programmes do harm. Some do good as well…” ^6^.

A central aspect of screening governance is therefore the weighing of potential harms against anticipated benefits. To that end, Wilson and Jungner emphasized the need for early, well-planned research to ensure that “scientific knowledge” rather than “folklore” guides screening practice ^1^. Continuing awareness of that need has encouraged the growth of clinical and epidemiological research capacity and the development of an international community of practice, including through professional societies and research associations (e.g. International Cancer Screening Network, International Agency for Research on Cancer Screening Group, International Society for Neonatal Screening). In concert with the evidence-based medicine movement, screening governance bodies have done much to advance this cause, developing systematic review processes, formulating explicit criteria to evaluate screening interventions, articulating evidentiary and methodological standards that accord with those criteria (e.g. types of harms, implementation considerations), and tailoring them to the specific populations or conditions ^7– 10^. Through these activities, and by actively fostering research and methodological refinement more generally, screening governance organizations have come in many cases to stand as exemplary proponents of evidence-based medicine.

### Enduring challenges

The adoption of clear evaluation criteria and robust evidential methods does not obviate the importance of value-based judgments and normative expectations in screening governance ^6, 11^. For instance, normative expectations inform decisions over what criteria are relevant when determining the balance of harms and benefits, as is evident from the way screening governance bodies have variously augmented or reinterpreted Wilson and Jungner’s original ten principles to include values such as informed choice and health equity ^12, 13^, while value judgements also inform how harms and benefits are weighted.

Further evidential challenges arise from the dynamics of screening practice itself. Screening governance bodies commonly influence evidence production through their decisions about what they consider to be adequate evidence (by type, quantity or quality). But decisions taken on the basis of such evidence may in turn lead to change in the evidential landscape. As Wilson and Jungner realized, implementing a screening intervention may severely constrain what kind of evidence can be ethically generated thereafter, as that intervention becomes the accepted standard of care ^1^. Moreover, once a particular screening test diffuses into practice, both the screening technology itself and the practices within which it is embedded rarely stand still. Screening bodies are developing strategies to enhance their capacity to deal with these shifting landscapes, for instance by developing new evaluation criteria and evidence review capacity. But in practice, they face a fundamental difficulty in adjudicating the balance of benefits and harms where screening programs already exist, and where testing technology or treatment standards continue to evolve relative to the evidence base that provided initial support for the screening intervention.

Finally, both the production and the interpretation of evidence involve social processes which can complicate the role that evidence plays in adjudicating between different policy and practice options. Screening governance organizations are not the only actors involved in generating and evaluating evidence or using it as a basis for decisions about screening practice. Practitioner organizations, public and private healthcare organizations and health technology assessment (HTA) bodies also evaluate screening interventions and issue guidelines and recommendations. The different positions that these organizations occupy within the wider social structures of screening, as well as the divergent professional and personal commitments they represent and the conflicting financial interests they may embody, can all lead to differences in how evidence is generated and interpreted ^11, 14– 16^. Providers and practitioners responsible for actually delivering screening tests and follow-up interventions may be faced with multiple guidelines based on different sources of evidence or standards of practice, leading to outcomes that may elude the scrutiny of screening governance organizations; ^17^ while adoption of guidelines depends upon many factors besides simply the quality of evidence or the clarity of the guidelines ^18^. Finally, clinical and public convictions about the value of screening may run contrary to screening governance organizations’ inclination, on the strength of new or existing evidence, to restrict or withdraw access to established screening interventions—witness for instance the controversy around the recommendation of the Swiss Medical Board to cease introduction of new mammography screening programs and to set a time limit on the ones that exist ^19^.

As a result of all these factors, screening governance cannot be reduced simply to rule-governed review of evidence. Rather, it must take account of the wider factors that inform the production and interpretation of evidence, and indeed what is actually considered to be evidence ^20^, as well as the way that implications drawn from that evidence are translated into recommendations for practice, and the way that those recommendations are implemented in the complex social world of medical knowledge and practice. Evidence is necessary but not sufficient: screening decisions may be evidence-based, but that does not mean that they are unequivocally determined by evidence ^21, 22^.

## Organizing population screening

In looking beyond the principles to the practice of screening, Wilson and Jungner appreciated that “the efficient practice of screening” may best be secured by coordinating “the whole screening operation, beginning with an appeal to the public, through screening tests, definitive diagnosis, treatment and follow-up, without breakdown in communications at any point”. Ideally, such organized initiatives would ensure not only that “persons found in need of treatment should be able to obtain it”, but that the same services were made available to “a whole community”. In light of these stipulations, Wilson and Jungner cautioned that it was better to invest in a limited number of programs “that are well planned and well executed” than to spread resources too thinly by pursuing “every feasible form of early disease detection and prevention.” Given inevitable funding constraints, moreover, the “benefit and effectiveness of the proposed programme” would need to be weighed against “other desirable objectives”. To support priority setting, Wilson and Jungner proposed an axis of national preparedness for screening with, on the low end, poor countries with limited organized care that might support “medical aid teams,” and on the other end, wealthy countries with highly organized care, “where integrated screening operations carried out under national arrangements may be expected”. This axis also accommodated the phenomenon of wealthy nations with health services that were neither integrated nor accessible, and which would house “expensive sporadic screening exercises … with poor communication and follow-up” ^1^.

Wilson and Jungner contended that organized screening operations are more effective than *ad hoc* or opportunistic screening both for maximizing benefits and minimizing harms. This contention now is widely accepted and supported by evidence. Organized screening programs have other virtues too. For instance, unlike *ad hoc* or opportunistic approaches where tests are simply offered to patients seeking clinical attention for other purposes, organized screening programs can monitor and respond to on-the-ground experience and uptake of screening. They can also provide a structure within which to pursue informed decision making and monitor and promote equity of access and outcome. Consequently, health insurance and care systems, both public and private, have increasingly come to favor organized screening programs as the best means to identify and recruit target populations, ensure relevant diagnostic and therapeutic follow-up, and monitor, evaluate and quality assure screening pathways ^23, 24^. In many cases, such programs are organised in concert with or in response to guidance from the competent screening governance organizations, providing screening governance organizations with a second critical mechanism by which to shape practice, in addition to systematic collection and review of evidence.

### Enduring challenges

The implementation of organized screening programs does not necessarily mean uniformity of provision, however. Even within a single program or jurisdiction, the organization of screening may not be consistent or comprehensive. Such internal variation impedes clarity about the pre-conditions of good outcomes. It also complicates efforts to compare performance and share insights *across* programs and jurisdictions. Indeed, what is meant by “organized” screening may itself vary markedly across jurisdictions, even for the same condition: programs in one jurisdiction may be organized only insofar as they involve systematic cohort notification or data registration, while those in another may organize the full screening pathway including failsafe mechanisms and quality assurance ^25^. Consequently, organized screening programs vary widely in the extent and nature of the services they provide.

This variation is not due solely to differences in “national preparedness”—the spectrum of organizational capacities and capabilities of national healthcare systems that Wilson and Jungner pointed to. Another key factor is the uneven organizational capacity and authority that screening governance organizations possess. Human and financial resources and sound management are needed to coordinate, quality assure and evaluate a screening pathway. Availability of data infrastructure, in particular, is often crucial if population-based screening programs are to be implemented, monitored and evaluated effectively ^26^. But screening governance organizations vary in their ability to mobilize such resources. They also differ in their powers to engage with health insurance and care systems that execute some or all of the steps in the full screening pathway. Screening governance organizations thus exist on a spectrum defined by their capacity to organize and coordinate programs. At one extreme are organizations that possess little more than a structured review function, with only a limited role in coordinating screening pathways or even in specifying how they should perform. At the other are organizations with the resources to mount pilot programs and conduct research including long term follow-up, and with the authority to guide and quality assure the conduct of screening and treatment by health care professionals and provider organizations. Even in the latter case, however, screening governance organizations are acutely dependent on the state of development of the relevant healthcare services and functions and on the extent to which diagnostic and treatment services are integrated, available and accessible. This poses a major constraint on screening governance organizations’ ability to design and direct effective programs. Under such circumstances, questions of equity and ethics may profoundly influence decisions about whether or not to establish screening programs in specific contexts ^27^.

Screening governance organizations are not typically in a position to determine how much resource or authority they possess. Indeed, what screening “is” in any one country reflects pragmatic realities in the allocation of responsibility for different practices. Such jurisdictional realities may produce expansive definitions of screening. The UK National Screening Committee, for example, brings a wide range of screening initiatives into one common programmatic framework, even as each screening program is executed independently across four distinct health systems. Elsewhere, however, screening governance organisations may have to work with more limited definitions of screening. In Australia, Italy and the Netherlands, for example, national screening programs forge a common approach across distinct states or regions for an intentionally narrow set of conditions, while other screening services are organised separately from these national programs, often on a regional basis.

Variation in the nature of organized screening programs is further complicated by the occurrence of opportunistic or *ad hoc* screening. Opportunistic screening may exist in the absence of organized effort, as is the case for PSA-based prostate cancer screening, which has been endorsed by few screening governance organizations. Alternatively, opportunistic or *ad hoc* screening may exist *alongside* organized screening programs, as is sometimes the case for cervical or breast cancer screening, especially where screening is pursued outside recommended guidelines (e.g. over or under recommended age limits, or at greater frequency). These overlaps may have serious implications for extant or potential organized screening programs. Even where screening governance organizations exercise strict control over organized programs, they are generally unable to control the *ad hoc* or opportunistic screening that may exist in parallel, nor can they avert the harms that are likely to occur where such screening occurs against express recommendations. Individuals who pursue opportunistic screening may not be able to secure timely access to the follow-on testing and treatment that an organized program can assure; or they may find themselves referred for follow-up in circumstances where it would not normally be justified; or they may be exposed to other unwarranted harms or costs. This may in turn have implications for screening governance organizations. In the absence of systematic data about the conduct and outcomes of competing or overlapping *ad hoc* and opportunistic screening, screening governance organizations’ ability to evaluate, quality assure and where necessary modify organized screening programs may be confounded and compromised ^25^.

Decisions about screening programs, then, are not necessarily just about whether or not to screen. They may be also about whether the initiation or termination of an organized approach will lead to improvements of one kind or another—be it in equality of access, or quality of testing and decision-making, or achieving a better balance of benefits and harms. Here too, value-based judgments and normative expectations are unavoidable. Decisions about where to focus organized screening effort and how to define the scope of screening programs do not simply follow from what is technically possible. Difficult trade-offs may be necessary, for example between the opportunity to increase access by under-served populations to existing screening interventions and the opportunity to establish new screening programs. Or circumstances may warrant the pursuit of less-than-optimal solutions: an organized and quality-assured program may increase benefit and reduce harm relative to an *ad hoc* or opportunistic program, for instance, even if the balance between benefit and harm would not justify the intervention in the first instance.

These organizational and institutional factors add a further dimension to the complexities and uncertainties we have already identified in relation to evidence review and evidence-based decision-making. As in the evaluation and weighting of evidence, judgements about how to organize screening programs have a strongly social dimension, colored by the play of professional and personal commitments and institutional and financial interests. And this too has implications for the work of screening governance organizations. In planning and overseeing organized screening programs, screening governance organizations do not only deal with evidence; they also need to engage with and take account of the concerns and interests of multiple stakeholders, from patients to practitioners to healthcare providers. Ultimately, the effectiveness of screening programs—their ability to deliver benefits while minimizing harms to individuals and populations—depends on how well these stakeholders can be encouraged or compelled to work together to a common end.

## Organizing screening governance

The challenge confronting screening governance organizations is thus not simply a technical one of assessing evidence and designing organized programs. It is also a question of how to coordinate social action, often under conditions of some uncertainty. How screening governance organizations achieve this is a vital aspect of the work of screening governance itself. And in this respect, much may depend on just how screening governance bodies are constituted, how they are situated in relation to other interested organizations and actors, what powers are vested in them, and what authority they wield.

Unsurprisingly, given the diverse political and healthcare settings in which they have emerged, screening governance organizations vary widely (see Box 2). Some are designated agents of government while others are independent committees of experts. Some are national with broad clinical scope; others are regional and focused on specific clinical areas. Some issue recommendations (variously to clinicians, health insurance and healthcare organizations, and government bodies) while others have formal authority, including the power to require reimbursement by insurers or action by health systems. To date, little effort has been made to understand how the social organization of screening governance affects the way screening itself is implemented, and with what consequences. However, we can identify a number of issues around which challenges may arise.

**Box 2. Screening governance organizations: some examples**

- The Netherlands: Health Council of the Netherlands (est. 1902) https://www.healthcouncil.nl/about-us/history
  - National government advisory body with very broad clinical scope
  - Advises Ministers and Parliament on matters of public health including population screening
  - Since 1997 charged with assessing applications for government licenses to undertake population screening
- The Netherlands: National Institute for Public Health and the Environment (1909) https://www.rivm.nl/en/about-rivm/rivm
  - National government agency with broad public health scope
  - Advises the Minister on the introduction and modification of screening programs
  - Designs, directs and coordinates national screening programs
- Canada: Task Force on Preventive Health Care (formerly Canadian Task Force on the Periodic Health Examination, est. 1976) https://canadiantaskforce.ca/about/history/
  - National government agency with broad clinical scope
  - Issues recommendations to clinicians
- USA: Preventive Services Task Force (1984) https://uspreventiveservicestaskforce.org/uspstf/procedure-manual-section-1
  - Independent committee of experts supported by government agency with broad clinical scope
  - High grade recommendations necessitate coverage by commercial insurers and states through Medicare and Medicaid
- USA: Advisory Committee on Heritable Disorders in Newborns and Children (2003) https://www.hrsa.gov/sites/default/files/hrsa/advisory-committees/heritable-disorders/about/ACHDNC-Discretionary-Charter.pdf
  - Statutory committee of experts appointed by the US Secretary of Health and Human Services
  - Advises government on screening for heritable disorders in newborns and children
- Ontario, Canada: Cancer Care Ontario (1997) https://www.cancercareontario.ca/en/cancer-care-ontario/about-us
  - Provincial government agency with scope for cancer control
  - Issues evidence-based recommendations to government
  - Plans, implements and operates Ontario’s organized cancer screening programs
- Ontario, Canada: Newborn Screening Ontario (2006) https://www.newbornscreening.on.ca/en/about-us
  - Provincial non-governmental agency with scope for newborn screening
  - Advisory Council established in 2010 reviews program quality and issues recommendations to government
- UK: National Screening Committee (1996) https://www.gov.uk/government/groups/uk-national-screening-committee-uk-nsc
  - National government agency with broad clinical scope
  - Advises on action by the health systems of the UK’s four countries.
- Australia: Medical Services Advisory Committee (1998) http://www.msac.gov.au/
  - Independent non-statutory committee established by the Minister for Health, with broad clinical remit
  - Appraises new medical services proposed for public funding, including national screening services
- Australia: Standing Committee on Screening (2001) http://www.cancerscreening.gov.au/internet/screening/publishing.nsf/Content/standing-committee-on-screening
  - Advisory body to national, state and territory governments on national cancer screening programs and issues, with an emerging advisory role in relation to newborn screening
- Italy: Osservatorio Nazionale Screening (2001) https://www.osservatorionazionalescreening.it/content/chi-siamo
  - Independent Agency mandated by National and Regional Governments
  - Monitors screening programs nationwide, and makes recommendations on new screening programs
- New Zealand: National Screening Advisory Committee (2004) https://www.nsu.govt.nz/about-us-national-screening-unit/nsu-advisory-groups/national-screening-advisory-committee
  - Advisory Group to Ministry of Health (who advises government) with broad clinical scope
  - Issues recommendations to the Ministry of Health for national population screening programs

### Enduring challenges

A key challenge facing screening governance organizations is how to negotiate the often conflicting interests and values of the different stakeholders involved in delivering the various elements of a screening program. Just whose voices get heard can significantly affect screening decisions, for instance over what conditions to include in newborn screening programs ^28^. Certain stakeholders may enjoy formal representation within screening governance organizations. More generally, screening governance organizations may be in a position to convene discussions with other organizations with overlapping roles related to screening activities—for instance the health insurance and healthcare organizations that offer *ad hoc* and opportunistic screening tests, or the professional associations whose guidelines on screening may conflict with those issued by screening governance organizations. As such, screening governance organizations may play a pivotal role by facilitating participation among the various scientists, clinicians and other healthcare providers whose research and care standards are critical to screening-relevant care, and whose decisions and practices can increase benefits or lead to harm.

Yet little has been done to examine how different screening organizations engage with scientists and clinicians, or how they involve them in evidence review and recommendation processes or the design and delivery of organized screening programs. We have only very patchy knowledge about which stakeholders are considered by different screening organizations to have a legitimate “stake” in deliberations over evidence or the design and delivery of organized programs, or about the processes they use to accommodate or respond to stakeholders’ views. Nor do we have any systematic insight into what types or processes of engagement are most effective at securing coordinated action around screening, under what circumstances, and with what outcomes or impacts. In principle, many screening governance bodies have the organizational depth and staying power needed to develop and support processes of engagement and deliberation that will potentially lead to significant improvements in the effectiveness of screening programs. In practice, however, we lack any overview of what different screening organizations actually do in this regard, let alone any insight into what works.

Questions of engagement do not extend solely to professionals and healthcare providers. They also potentially extend to the individuals and populations who undergo screening, and who stand to experience both the benefits and the harms that screening may deliver. These people, as much as the professionals, determine the legitimacy of screening interventions, be it by bringing political pressure to bear on the relevant authorities or simply by consenting or declining to participate in screening. Screening governance organizations are often active in communicating their conclusions and rationales widely and for diverse publics ^29^, and there is evidence that active engagement may have a significant impact on public attitudes towards screening ^30^. They may also have the capacity to provide opportunities for public input, for example through public membership of review committees, or stakeholder input on draft recommendations. This too raises questions about exactly whose views should be taken into account: just those individuals identified as having a positive screening test result, or all members of a target population, or entire national populations? Public attitudes regarding the desirability of screening may depart markedly from expert evaluations based on evidence of harms and benefits, and may differ between those affected and those unaffected by particular diseases ^31, 32^. This raises profound ethical questions about who should be involved in screening policy decisions, and what methods should be adopted to elicit or inform their views. As yet, however, we have no systematic overview of what different screening bodies do to engage participants or publics, or to what effect. Given growing expectations of patient involvement in the planning and delivery of healthcare services, however, much could be gained from more concerted reflection on what public and patient engagement can contribute to screening governance.

## Conclusions

Wilson and Jungner’s 1968 report remains a critical touchstone in efforts to adjudicate and balance the benefits and harms of population screening. Half a century on, their principles and their attention to practices have informed the emergence of a range of governance organizations employing sophisticated governance mechanisms, including structured evidence review processes to determine whether and how to screen, and programmatic efforts to maximize benefits and minimize harms. At a meeting of international screening experts convened in the summer of 2018 to reflect on Wilson and Jungner’s contribution to the governance of screening, participants highlighted that organized oversight and accountability for population screening remains crucial. Only through coordinated governance procedures can recommendations about screening invoke clear criteria, robust systems of evidence review and deliberation, and transparent processes of communication and public engagement. Such procedures are equally important to ensure that screening practices address defined populations, provide integrated screening and care pathways, possess robust data capacity, and ensure detailed quality assurance and long-term follow up.

At the same time, the workshop participants expressed a desire for more sustained reflection on how screening governance works in different jurisdictions, and how the organization and implementation of governance procedures impacts on screening recommendations, programs, and ultimately outcomes. There is a perceived need for greater awareness and understanding of these critical issues, including how screening governance bodies are constituted, what powers they possess, how they exercise those powers, how they engage with stakeholders including healthcare providers and publics, and how all these factors affect decision-making processes and the outcomes of those decisions.

That is not to suggest that there should be greater standardization or homogeneity of screening governance arrangements across jurisdictions. The social, political and economic circumstances under which screening is enacted vary enormously, and governance arrangements need to remain attuned to local circumstances. But learning across borders can still be valuable. One way to pursue such learning would be through research that characterizes and compares screening organizations and governance arrangements across clinical conditions and across countries. But even in the absence of such research, enhanced opportunities and incentives for informal exchange and engagement between different screening governance organisations, including sharing experiences and insights into best practices, could be beneficial. Indeed, in such complex arenas of policy and practice, holistic, experience-based apprehension of how different organizations have adapted to deal with the contingent circumstances under which they operate may be a more fruitful means of adaptive policy learning than formalised comparison ^33, 34^. Academic research can do much to facilitate policy learning. But even in the absence of such research, informal exchange and sharing of experiences between screening governance organizations and cognate expert bodies has the potential to deliver invaluable insights into the social as well as the technical aspects of governance. Those involved in the work of screening governance may learn much from observing how sister organizations in other jurisdictions exercise the authority and powers vested in them, how this relates to variation in the scope and practice of screening, how they engage the interests of practitioners and publics, and how effective governance can ensure the legitimacy and public acceptability on which screening ultimately depends.

## Data availability

### Underlying data

No data is associated with this article.
