# Supplementary material for: Half a Century of Wilson & Jungner: Reflections on the Governance of Population Screening
Source: Wellcome Open Res. 2020 Aug 17;5:158. Originally published 2020 Jul 6. [Version 2] doi: 10.12688/wellcomeopenres.16057.2 (PMC7468564; doi:10.12688/wellcomeopenres.16057.2)
Supplement: Peer review history

# Peer review: Reviewer response for version 2

**DOI:** 10.21956/wellcomeopenres.17834.r40173

**Published:** 2020-09-30

**Authors:** Alex Faulkner

**Affiliations:** 1 University of Sussex, Sussex, UK

The Open Letter identifies an interesting and potentially important neglected issue in medical screening – the constitution, behaviour and operation of ‘screening governance organisations’ (SGOs). A notable selection of experts in public health, screening policy, practice, medical history and sociology contribute to the Letter. The approach taken usefully acknowledges what could be called the ‘sociopolitical’ aspects of screening policies and their production, hence providing a complement and corrective to approaches that focus exclusively on the historical ‘principles and practices’ of screening and policies which are taken, explicitly at least, to be based only on scientific and medical evidence and the weighing of benefits and harms defined through public health and narrow ‘evidence-based medicine’ lenses. The review that the workshop-based Letter undertakes, as the authors claim, could provide the basis for programmes of mutual learning amongst such organisations and the sharing of good practice, if such practice can be identified in a transportable way. A number of points of potential improvement and current lack of data are usefully identified, for example information about the involvement of different stakeholders in SGOs’ framing of ‘what counts’ as evidence, appraisal work and decision-making. The pointers to a research programme that are set out should be taken seriously by various potential funding organisations internationally.

 The paper usefully maps out the range of SGO’s from those with limited resource carrying out ‘structured reviews’ to well endowed organisations able to carry out long term follow-up research and build infrastructures to provide guidance and implementation powers. Likewise, some SGO’s focus on a narrow range of medical conditions, while others are set up with a broad-ranging remit.

 The role of patient and civil society organisations, NGOs etc., known to have had a contentious impact on some screening programme policies worldwide, is not addressed head-on in the paper, rather, such issues are referred to in terms of the language of ‘public and patient engagement’. The well-known frequent divergence of values and views between public health professions and certain public or patient groups is noted. In this respect the authors rightly note the crucial importance of public trust and the societal credibility of screening programmes. It would be useful to understand more about how SGOs take strategic measures to address this issue, which is key in the current times of grappling with governance of scientific uncertainty. To what extent, for example, do different SGOs in different local situations adopt ‘precautionary’ approaches?

 It’s notable that screening trials per se as a form of evidence-production are not referred to explicitly, though it is well known that very expensive international population screening outcome trials have been conducted (for example in prostate cancer screening), with inconclusive results. It will be interesting to learn more about the relations between SGOs and public sector research funders and ‘health services research’ organisations, both clinical and academic, in this respect.

 The paper provides a useful selective list of Western SGOs with short notes of their roles. It will be useful to see more detail of the constitution of such organisations in due course. The mention of Wilson & Junger’s 1968 WHO reference to ‘medical aid teams’ in developing countries suggests that it will be interesting also to expand this to a consideration of low and middle income countries, where importation and adaptation of Western governance models is a known phenomenon in many fields.  

 The focus of the paper on socio-political and economic factors in SGO constitution and behaviour suggests that investigation of the relationships between SGOs and national political and economic cultures and infrastructures will be a productive area in order to try to assess the likely constraints on mutual learning and sharing of perceived good practice. This might also be a consideration in following up the authors’ promising suggestion that SGOs can hold a pivotal role mediating between scientists, clinicians and others in developing screening governance in multi-stakeholder contexts.

 Finally, one can wonder what should be defined, in different national and other contexts, as the *societal* governance outcomes of SGOs, aside from the already contested and difficult assessment of medical harms/benefits?

 While the analysis presented in the paper is that of a generic and valuable overview, given the multiple authors and aim to map the SGO phenomenon in an essentially novel way, it opens up a wide range of further questions worthy of exploration through research, especially more detailed comparative analysis of contrasting SGO governance regimes.

Does the article adequately reference differing views and opinions?

Yes

Are all factual statements correct, and are statements and arguments made adequately supported by citations?

Yes

Is the Open Letter written in accessible language?

Yes

Where applicable, are recommendations and next steps explained clearly for others to follow?

Yes

Is the rationale for the Open Letter provided in sufficient detail?

Yes

Reviewer Expertise:

Medical Sociology and 'Science & Technology Studies' (STS) study of  scientific medical and healthcare innovations; HTA study of health technologies including screening programmes.

I confirm that I have read this submission and believe that I have an appropriate level of expertise to confirm that it is of an acceptable scientific standard.

# Peer review: Reviewer response for version 2

**DOI:** 10.21956/wellcomeopenres.17834.r39976

**Published:** 2020-09-02

**Authors:** Nigel G. Laing

**Affiliations:** 1 Centre for Medical Research,Harry Perkins Institute of Medical Research, QEII Medical Centre, University of Western Australia, Nedlands, WA, Australia

I would just like to thank the authors for their responses to my comments and suggestions and I am glad the authors found them useful.

Does the article adequately reference differing views and opinions?

Partly

Are all factual statements correct, and are statements and arguments made adequately supported by citations?

Partly

Is the Open Letter written in accessible language?

Yes

Where applicable, are recommendations and next steps explained clearly for others to follow?

Yes

Is the rationale for the Open Letter provided in sufficient detail?

Yes

Reviewer Expertise:

Human molecular genetics, molecular diagnosis, reproductive carrier screening

I confirm that I have read this submission and believe that I have an appropriate level of expertise to confirm that it is of an acceptable scientific standard.

# Peer review: Reviewer response for version 1

**DOI:** 10.21956/wellcomeopenres.17613.r39421

**Published:** 2020-07-20

**Authors:** Nigel G. Laing

**Affiliations:** 1 Centre for Medical Research,Harry Perkins Institute of Medical Research, QEII Medical Centre, University of Western Australia, Nedlands, WA, Australia

This Open Letter is based on a Workshop organised in 2018 to commemorate 50 years of the Wilson and Jungner criteria.

 I agree with Professor Cornel that there is an over-emphasis on cancer screening and too little discussion on genetic screening. I feel there is insufficient discussion of how the original 1968 Wilson and Jungner criteria do not work for genetic screening as has been discussed by others:

 Reference 12 - Andermann, A., Blancquaert, I., Beauchamp, S. & Dery, V. Revisiting Wilson and Jungner in the genomic age: a review of screening criteria over the past 40 years. *Bull World Health Organ* **86**, 317-9 (2008).

 But also:

- Grosse, S.D. *et al.* Population screening for genetic disorders in the 21st century: evidence, economics, and ethics. *Public health genomics* **13**, 106-15 (2010).
- Andermann *et al. *(2011 ^1^). 

The insistence in Wilson and Jungner 1968 on a treatment being available means that genetic screening does not fit the criteria. For example, strict adherence to the 1968 Wilson and Jungner criteria has been used to block newborn screening for Duchenne muscular dystrophy, because there was no cure for Duchenne muscular dystrophy. This has led to who knows how many second, third or more affected boys being born to couples who might have wanted to avoid having more than one affected son.

 Similarly, there is insufficient discussion that the 1968 Wilson and Jungner criteria do not fit with current public opinion or government thinking on reproductive carrier screening.

 Reproductive carrier screening does not fit with 1968 Wilson and Jungner in that again there is no treatment for many of the diseases now screened for. The benefit is not to a patient but to prospective parents. In relation to public opinion, from my Group’s work, we know that two-thirds of Western Australians wish access to reproductive carrier screening, while only 10% do not want to access it (Ong *et al., *2018 ^2^).

 1968 Wilson and Jungner does not fit with Government thinking in Israel, which is running a nationwide panethnic carrier screening program (Zlotogora *et al.*, 2016 ^3^), or Australia, where the Federal Health Minister has called for reproductive carrier screening to be made available in the future free to all Australians who want to use it (Delatycki *et al.*, 2019 ^4^ and Casella, 2020 ^5^).

 I just wish that articles about Wilson and Jungner criteria, especially articles on governance of screening, would strongly acknowledge that strict adherence to 1968 Wilson and Jungner is no longer viable, and that every “screening governance organization” needs to use modified Wilson and Jungner criteria in 2020.

 This Open Letter however is based on a specific workshop, so if these things were not discussed at the Workshop, then perhaps they cannot be included in the Open Letter. It may then be up to others to publish counter arguments at a later date. 

 In Australia, as well as the Medical Services Advisory Committee (MSAC) ( http://www.msac.gov.au/), which advises the Federal Government on which items the Federal Government should fund for the population, there is the Standing Committee on Screening: ( http://www.cancerscreening.gov.au/internet/screening/publishing.nsf/Content/standing-committee-on-screening) (SCOS). The SCOS advises the Australian Health Ministers Advisory Council (AHMAC) on which conditions should be screened for. AHMAC includes the Federal and State Health Ministers. Newborn screening in Australia is not funded by the Federal Government, but by the Health Departments of the individual States and Territories. Newborn screening is therefore not under the Federal Health Department’s jurisdiction, and MSAC is therefore irrelevant to newborn screening in Australia.

 Minor comment, under “Methods” and “An expert policy workshop”, which are the three lead authors? The first three authors or the last three authors or a mixture? Would it be clearer to simply give their names?

Does the article adequately reference differing views and opinions?

Partly

Are all factual statements correct, and are statements and arguments made adequately supported by citations?

Partly

Is the Open Letter written in accessible language?

Yes

Where applicable, are recommendations and next steps explained clearly for others to follow?

Yes

Is the rationale for the Open Letter provided in sufficient detail?

Yes

Reviewer Expertise:

Human molecular genetics, molecular diagnosis, reproductive carrier screening

I confirm that I have read this submission and believe that I have an appropriate level of expertise to confirm that it is of an acceptable scientific standard, however I have significant reservations, as outlined above.

## References

[1] : Guiding policy decisions for genetic screening: developing a systematic and transparent approach. *Public Health Genomics*.2011;14(1) : 10.1159/000272898 9-16 10.1159/000272898 20051674

[2] : Measuring the impact of genetic knowledge on intentions and attitudes of the community towards expanded preconception carrier screening. *J Med Genet*.55(11) : 10.1136/jmedgenet-2018-105362 744-752 10.1136/jmedgenet-2018-105362 30068663

[3] : The Israeli national population program of genetic carrier screening for reproductive purposes. *Genet Med*.2016;18(2) : 10.1038/gim.2015.55 203-6 10.1038/gim.2015.55 25880436

[4] : Preconception and antenatal carrier screening for genetic conditions: The critical role of general practitioners. *Aust J Gen Pract*.48(3) : 10.31128/AJGP-10-18-4725 106-110 10.31128/AJGP-10-18-4725 31256466

[5] : Mackenzie’s Mission. *Allen and Unwin*.2020;187-196 Reference source

# Response

**Published:** 2020-08-10

**Authors:** Steve Sturdy

**Affiliations:** University of Edin, UK

Like Prof Cornel (reviewer 1), Prof Laing notes that more workshop participants came from the field of cancer screening than from genetic screening. As both reviewers observe, different kinds of conditions, and different kinds of tests, entail different assessments of potential harms and benefits; indeed, every proposed screening intervention needs to be evaluated on its particular merits. However, our aim in the workshop and in this open letter was to step back from specifics, and to explore issues common to the experience of all the participants, which we believe need to be taken into account when considering governance arrangements for screening interventions in general. Besides experts from cancer screening and from organisations with a comprehensive purview of all kinds of population screening, the workshop included several participants with particular expertise in screening for genetic disorders. All participants contributed fully to the proceedings and to the drafting and finalization of the open letter, and all approved the views expressed. We are therefore confident that we have succeeded in our aim of identifying issues of relevance to the governance of all forms of population screening modalities across a range of jurisdictions.

Regarding Prof Laing’s comment that “strict adherence to 1968 Wilson and Jungner is no longer viable”: We should stress that our article does not advocate strict adherence to the precise 1968 principles. On the contrary, we point out the complexities and uncertainties inherent in weighing the harms and benefits of a growing diversity of screening interventions, and the ways that the 1968 principles have therefore been variously reinterpreted and augmented. Our intention was to invoke the spirit as much as the letter of the Wilson and Jungner reflections on screening governance, and to highlight in particular their often-neglected observation that practice is as important as principles in ensuring that screening delivers a positive balance of benefit over harm. Space did not permit us to pursue this insight down to the level of individual screening interventions. Instead, we sought to draw general reflections on how screening governance might best be arranged in order to ensure that all kinds of screening interventions, including genetic as well as cancer screening, deliver appropriate benefits to the populations they are designed to serve. There will be far more to say about just what this might entail for particular interventions and particular circumstances – and we are delighted that our reviewers have already begun to pursue that conversation.

On a point of accuracy, Prof Laing noted a deficiency in our summary, in Box 2, of Australian screening governance arrangements. We have now amended that entry with the addition of the Standing Committee on Screening.

On a specific textual point, Prof Laing helpfully notes that it is unclear whom we refer to when we talk of the “lead authors” of this open letter. We have now replaced that phrase with “Sturdy, Millar and Hogarth”.

# Peer review: Reviewer response for version 1

**DOI:** 10.21956/wellcomeopenres.17613.r39422

**Published:** 2020-07-08

**Authors:** Martina C. Cornel

**Affiliations:** 1 Department of Clinical Genetics, Section of Community Genetics, Amsterdam Public Health research institute, VU University Medical Center, Amsterdam, The Netherlands

The paper presents a report based a workshop organized in 2018. It provides reflections on the governance of population screening programmes, which indeed is very topical and much needed. Governance of screening in many countries faces challenges of a changing landscape in terms of technical possibilities for testing and treatments becoming available, while resources are limited.

 The participants were chosen from personal contacts and snowballing. This seems to have led to an overrepresentation of the Cancer Screening field, as well as Anglophone countries, and an underrepresentation of international umbrella-organizations and “genomic” screening (See Andermann 2008). Reproductive screening and neonatal screening may raise different issues in terms of evidence and ethics. 

 As a separate issue, “governance” may be considered a multi-step procedure where some stakeholders are involved in the (scientific) weighing of the evidence before a decision is made whether or not to start a screening, while other stakeholders are involved in the decision making on ethical and economical aspects (Ministry of Health, Parliament). Is “governance organization” a correct framing, or should rather the different steps in governance be studied in future analyses? Is the governance different for considering expansion of screening vs. responsible implementation of current screening programs?

Does the article adequately reference differing views and opinions?

Partly

Are all factual statements correct, and are statements and arguments made adequately supported by citations?

Yes

Is the Open Letter written in accessible language?

Yes

Where applicable, are recommendations and next steps explained clearly for others to follow?

Yes

Is the rationale for the Open Letter provided in sufficient detail?

Yes

Reviewer Expertise:

genetics & public health

I confirm that I have read this submission and believe that I have an appropriate level of expertise to confirm that it is of an acceptable scientific standard.

# Response

**Published:** 2020-08-10

**Authors:** Steve Sturdy

**Affiliations:** University of Edin, UK

Prof Cornel rightly observes that our method of recruiting participants meant that English-speaking countries were better represented than others. In consequence, we acknowledge that the views shared in the workshop and in the open letter focused mainly — though not exclusively — on the experiences of Western/English speaking countries. We hope that this open letter will help to stimulate further conversations covering a wider range of communities and jurisdictions.

Prof Laing (reviewer 2) raises the same question as Prof Cornel regarding differences between cancer screening and genomic, antenatal and newborn screening. We therefore discuss this question in our response to Prof Laing’s comments.

On the question of governance as a multi-step procedure: a distinction can be drawn between scientific assessment of evidence on one hand and ethical, economic and social aspects of decision making on the other. However, one of the key points arising from our deliberations was that in practice, collection and evaluation of evidence regarding the establishment and expansion of screening unavoidably involves ethical, economic and/or social considerations about responsible implementation, just as much as does the assessment of existing screening programs. That is not to say that there is no value in organising screening governance in a multi-step fashion, to reflect the different issues that may be involved in different kinds of decision making. But any decision to do so will need to be taken on pragmatic grounds, considering what is practical and appropriate under the prevailing circumstances, and not on the assumption that some aspects of governance are purely technical or scientific, while others are ethical or social.
